# Bureau of Animal Industry

**Published:** 1903-03

**Authors:** 


### BUREAU OF ANIMAL INDUSTRY.

---

PROMOTIONS OF VETERINARIANS DURING THE YEAR 1902.

From $2250 to $2500: Dr. John R. Mohler, Chief of Pathological Division, Washington, D. C.

From $2000 to $2250: Dr. O. E. Dyson, inspector in charge, Chicago, Illinois.

From $1400 to $1600: Dr. Joseph B. Clancy, National Stock Yards, Illinois (placed in charge of station).

From $1400 to $1500: Drs. Leslie J. Allen, Oklahoma City, Oklahoma; Thomas Castor, Trinidad, Colorado; C. H. Davies, Kansas City, Kansas; Charles M. Day, Sidney, Iowa; H. H. George, Kansas City, Kansas; A. A. Holcombe, Aurora, Illinois; G. A. Johnson, Sioux City, Iowa; W. H. Smith, Jr., Chicago, Illinois; Walter J. Stewart, Chicago, Illinois; B. P. Wende, placed in charge, Buffalo, New York.

From $1200 to $1400: Drs. H. M. Ball, Buffalo, New York; H. M. Batchelder, South Omaha, Nebraska; E. L. Bertram, Chicago, Illinois; Herbert Caldwell, Milwaukee, Wisconsin; E. T. Davison, placed in charge, Rushville, Nebraska; Arthur R. Glaisyer, Spokane, Washington; Joseph M. Good, inspector in charge,

Dayton, Ohio; John S. Grove, Kansas City, Kansas; H. J. Hammond, Amedee, California; John J. Hayes, Chicago, Illinois; Daniel S. Hays, Boston, Massachusetts; R. W. Hewett, Philadelphia, Pennsylvania; John B. Hopper, New York City; George T. Irons, South St. Paul, Minnesota; Raymond Johnson, South St. Joseph, Missouri; Louis A. Klein, Fort Worth, Texas; Clarence Loveberry, inspector in charge, Seattle, Washington; Albert Long, Boston, Massachusetts; Frank C. McCurdy, South St. Joseph, Missouri; Monroe B. Miller, New York City; George Byron Morse, Washington, D. C.; B. W. Murphy, Jr., South St. Joseph, Missouri; Wesley N. Neil, inspector in charge, Waterloo, Iowa; Isaac W. O'Rourke, Reno, Nevada; James L. Otterman, Kansas City, Kansas; Charles F. Palmer, Indianapolis, Indiana; John O. F. Price, National Stock Yards, Illinois; Henry Roome, Sioux City, Iowa; H. R. Ryder, Buffalo, New York; William G. Shaw, Nogales, Arizona; Daniel G. Shumway, South St. Paul, Minnesota; John A. Sloan, South St. Joseph, Missouri; Herbert M. Smith, Louisville, Kentucky; Nathaniel B. Smith, Billings, Montana; F. L. Stevens, Boston, Massachusetts; A. W. Swedberg, Kansas City, Kansas; Joseph J. Thackaberry, New York City; Norris L. Townsend, Helena, Montana; A. H. Wallace, San Antonio, Texas; Thomas White, Douglas, Wyoming; Alexander E. Wight, Boston, Massachusetts; Warner W. Worcester, Des Moines, Iowa.

---

ANTIRABIC INOCULATION IN COLTS. Among a lot of 47 colts Kurtz and Anjezky, of Budapest, found two which showed characteristic symptoms of rabies, and died in a few days. Examination showed that in seven others small scars were found on the body; these were suspected as being infected from bites, although there was no other positive evidence. Two days afterward one of the seven died of rabies.

The 44 colts were submitted to antirabic inoculation with a virus prepared at the Pasteur Institute of Budapest after the method of Hoegyes. Three inoculations were made under the skin of the neck at intervals of five and two days. There was no hyperthermia nor any other indication of any reaction, and the animals remained in perfect health, and six months afterward no rabies had recurred.

This is the first time antirabic inoculations have been made in veterinary medicine, and they seem to be efficacious when made in time.—*Deutsche Thier. Wochen.*

